# Gender differences in multi-employee gift exchange with self-reported contributions

**DOI:** 10.1371/journal.pone.0238236

**Published:** 2020-09-01

**Authors:** Veronika Grimm, Holger A. Rau, Simeon Schächtele

**Affiliations:** 1 University of Erlangen-Nuremberg, Nuremberg, Germany; 2 University of Göttingen, Göttingen, Germany; 3 Inter-American Development Bank, Washington, DC, United States of America; Middlesex University, UNITED KINGDOM

## Abstract

Gender-wage gaps are an important phenomenon on labor markets. They can possibly be caused by the institutional framework. This question is addressed in this paper. When only joint output can be observed in team production, individuals may submit self-reports of their contribution to a principal. In a multi-employee gift exchange experiment, we study how men and women behave differently with and without such self-reports. We cannot reject that self-reports left the overall efficiency of the gift exchange interaction unchanged, but detect notable gender differences. Women reported similar effort levels as men, but contributed significantly less. The difference in contributions led to a significant gender gap in wages, depending on gender group composition. These effects were only present when participants did not know each other’s gender, however. When instead gender was observable, the behavior of men and women converged. The results suggest that parts of wage gaps may be related to different behavior within incomplete contract and imperfect information environments, depending on details of the informational context.

## Introduction

Gender-wage gaps are a severe problem on labor markets, which exists in many industries across countries [1, 2]. Income differentials between women and men may arise due to several reasons. First, supply-side issues may matter, i.e., women may prefer to work part-time, to choose different educational tracks than men, or may select into occupational fields that are characterized by a lower average income [3]. Experiments highlighted that gender differences in risk preferences and competitiveness may explain this behavior [4, 5]. Second, demand-side issues matter, i.e., women may be discriminated in hiring decisions [6], receive lower wages [7] and worse evaluations [8, 9] than men. Gender gaps may also occur as a consequence of interaction effects between demand-side and supply-side issues. Here, institutional factors play a crucial role, as they can facilitate the emergence of gender-wage gaps. For instance, bonus systems may lead to discriminatory behavior. That is, [7] report that female employees receive lower bonuses when they behave less competitively than men. Female employees may refrain from applying to internal promotion programs when they anticipate that they might be discriminated by employers [10].

To shed more light on the possible consequences of labor-market institutions on the gender-wage gap, we focus on the case of self-reports. Here, employees in teams have to report their effort to their employers. This scenario is relevant, as work is increasingly carried out in teams. It is common in such situations that only the joint output of a team can be observed precisely, but not the individual contributions. An employer may find it difficult to adequately compensate employees for their individual contributions. Thus, free riding and horizontal inequity among workers may ensue. In such a situation, employees sometimes submit self-reports of their contributions, either as part of an employer-mandated performance appraisal process or informally in an attempt to influence wages in their favor. We believe that self-reporting institutions may be important sources, which drive gender-wage gaps. The reason is that they share *two* characteristics where gender differences in preferences may play a crucial role. First, employees have to report outcomes, which are not perfectly observable by the employer. In this respect, it is possible to report untruthfully. Experimental economics repeatedly showed that men behave more dishonestly than women [11–13]. Second, self-report institutions build on trust, as employees move first when they exert effort. They face a risky situation not knowing whether the employer will reward the effort. Experimental economics highlighted that women behave more risk-averse than men [4] and female first movers trust less than male ones [4, 14, 15]. Thus, it is likely that a gender-wage gap arises in such a scenario, as a consequence of men exerting higher effort and inflating their reports to a higher extent than women. Research in experimental economics also showed that gender gaps are more pronounced when women know that their competitors are men [16]. Therefore, self-reporting institutions where the gender of co-workers is known are interesting institutional settings to analyze gender differences, which may stimulate gender gaps.

We study this situation experimentally within a multi-employee gift exchange context, focusing on the interaction of self-reporting and gender. Gift exchange and incomplete-contract environments in which reciprocity-based mechanisms work as enforcement device are an important element of the business environments within companies [17, 18]. Related research has focused on the robustness and limitations of gift exchange, notably the presence of multiple agents [19–21]. We add to this literature by studying self-reports and gender differences of employees in settings with multi-employee gift exchange. Our experimental design is closely related to the reverse gift exchange game in [20]. However, a crucial difference in our design is that individual contributions are not observed. In our experiment, a principal (‘employer’) chooses wages after observing the revenue generated from the costly effort contributions. The effort contributions are chosen simultaneously and separately by two agents (‘employees’). The lack of full observability of individual contributions has the consequences that co-workers may free ride and that horizontal inequity may arise for employees. The lack of observability of effort is arguably an important element of many team production settings. Beyond implementing this imperfect information feature in our baseline treatment, the between-subject treatment variation we study, consists of several treatments, where we study the effects of self-reporting efforts and known gender of the subjects. That is, in a second treatment, employees additionally submit a ‘cheap-talk’ self-report of their contribution to the employer. In both the first and second treatment, the participants were not aware of each other’s gender. The third treatment was identical to the second except that all participants were made aware of each other’s gender. Here, we test whether knowing the gender of the co-worker affects employees’ behaviors. This is motivated by the literature, which showed that stereotypical beliefs may matter in compensation decisions [6, 7]. Furthermore, there is evidence, which highlights that knowing the gender of opponents may affect subjects’ competitiveness [16]. In this treatment, we can analyze whether knowing the gender of the employees leads to discrimination from the employer’s side. We can also analyze whether the information on a co-worker’s gender affects exerted and reported effort levels. The three treatments we study, were balanced with regard to employee gender, facilitating the analysis of gender differences within and between treatments.

Our main results are as follows. We find notable gender differences when participants were not aware of each other’s gender. In this case, women contributed less than men, in particular when efforts were self-reported. Interestingly, we observed that both genders reported similar effort levels. The difference in actual contributions nevertheless led to a significant gender-wage gap, depending on the gender composition of employees. That is, in mixed gender groups, the non-observability of individual contributions implied similar wages for both employees. By contrast, in homogeneous gender groups, the gender difference in contributions translated to lower wages in all-female groups, which contributed less than all-male groups. Our treatment with gender observability showed significant time dynamics. That is, we find that men and women converged to similar levels of efforts, self-reports, and wages. The reason is that male employees increased free-riding behavior over time. Our study contributes to the analysis of the impact of labor-market institutions for the emergence of gender differences. We highlight that such scenarios may stimulate gender gaps, since female employees are lacking trust in these uncertain situations. Moreover, we contribute to the literature on multi-employee gift exchange games when individual contributions are unobservable. In several ways, our baseline treatment is comparable to the equal wage treatment of [20]. That is, wages cannot be conditioned on individual efforts and gift exchange efficiency is found to be limited. In this context, the effects of self-reporting have only been studied by [22] who find a negative effect on gift exchange efficiency in a slightly different game. We concentrate on gender differences, as they have been neglected in these scenarios. For multi-employee settings, our study reveals previously undocumented gender differences and highlights the impact of wage dynamics on gender-wage gaps. The finding of lower female contributions in treatments without gender observability may be related to prior findings of women being less trusting [23, 15] and to findings that women are relatively more focused on equality rather than efficiency [24–26]. If women do not trust in trust games this yields the outside payoff, which is characterized by equal payoffs. At the same time, this payoff is not efficient, as the payoff would be multiplied when subjects trust in the trust game.

Our experimental findings are interesting, since the differences in contributions of men an women point to a source of wage differentials that would be difficult to detect in naturally occurring data. Because these differences depend on the gender group composition, our results also relate to an emerging research, focusing on the interaction of gender differences and group composition [27]. Moreover, our treatment with gender observability highlights that male contributions decrease over time when gender is observable. Therefore, we demonstrate how details of the informational context and group composition affect the degree of heterogeneity between employee groups. The results have potential implications for companies that ponder introducing self-report mechanisms or that encounter self-reporting employees. They should have in mind that this may facilitate gender gaps.

## Methods

Our experimental design builds on the multi-employee reverse gift exchange game of [20]. In this game, two employees are matched with an employer. The employees simultaneously and independently choose effort levels between 1 and 10, with associated convex costs as displayed in Table 1. As in [20], the sum of efforts was multiplied by 10 and generated revenue for the employer. After observing revenue, the employer could pay each employee a wage between 0 and 100. Wages were not restricted to the amount of revenues, so employers (like employees) could incur losses. In other words, payoffs were $\pi_{i}^{A}=w_{i}-c\left(e_{i}\right)$ for an employee (*i* ∈ 1, 2) and *π*^*P*^ = 10 ⋅ (*e*_1_ + *e*_2_) − (*w*_1_ + *w*_2_) for the employer. As marginal revenue is always above marginal costs of efforts, full effort provision maximizes group surplus (“is efficient”). This was true in all three treatments we now describe.

**Table 1:** Costs of effort.

Level of effort *e*_*i*_	1	2	3	4	5	6	7	8	9	10
Cost of effort *c*(*e*_*i*_)	0	1	2	4	6	8	10	13	16	20

The main difference between our experimental design and the one by [20] centers around the observability of individual efforts. Specifically, in all of our three treatments, employers could only observe the sum of efforts, not the individual components. In our baseline treatment ‘No Report’ (*NR*), employers observed the revenue generated and then chose individual wages. As there was no information to base wage differentiation on, employers in this treatment are effectively driven into paying equal wages for potentially unequal efforts, somewhat akin to the equal wage treatment in [20]. In this treatment of [20], employers observed individual efforts but could not pay different wages. By applying the *NR* treatment, we can test whether the uncertainty that employees have to move first and that individual effort cannot be observed, is sufficient for male and female contributions to differ.

In the two remaining treatments, an additional stage between effort and wage choice allowed employees to send a signal about their effort contribution to the employer. In this stage, employees had to report an effort level between 1 and 10 to the employer. They did so simultaneously and without knowing the co-worker’s exerted effort. It was common knowledge that the report had no cost and no direct effect on revenue. The employer then observed total revenue and the reported effort of each employee before choosing wages. In treatment ‘self-report’ (*SR*), these self-reports of effort are the only difference to the baseline treatment *NR*. This treatment comparison therefore allows us to isolate the effects of self-reporting labor-market institution for the emergence of a gender-wage gap. To study whether employees change their behavior when knowing the gender of the co-worker and whether they might be discriminated against, we ran a further treatment: ‘self-report Gender’ (*SRG*). This treatment analyzes stereotypical effects when revealing gender. It is similar as treatment *SR*. However, the main difference to *SR* is that each participant was in addition provided with information about the gender of the other members of her group. Such gender information was provided in a salient manner, including the use of different colors for the two genders on the computer screens. In this respect, each employee was prompted to a box, where it was stated: “gender of the other employee: man (woman); gender of the employer: man (woman).” Additionally, we colored the word “man” (“woman”) in blue (red). The employer was also informed about the gender of each employee. Here we said: “In this round a man (woman), employee 1; and a man (woman), employee 2 worked for you. Again we colored the word “man” (“woman”) in blue (red). The comparison between treatments *SR* and *SRG* therefore allows us to discern the effects of gender observability in a team production setting with self-reporting. In this respect, we can study whether male or female employees adjust effort levels and self-reports, when knowing their co-worker’s gender. Moreover, we can study the reactions of employers. Thus, we can analyze whether discrimination plays a role in the wage-setting process.

In each treatment, the described game was played 24 times, with anonymous random re-matching of participants within matching groups of nine participants. The roles of employee and employer were kept throughout the experiment. After each period, employees learned their own wage as well as their co-worker’s wage and provided effort (but not the report). Employers were not provided new information at this stage (e.g. as regards individual efforts) and instead told their period and cumulative payoff.

The experimental instructions were framed in a labor market context of employers and employees. An English translation of the instructions can be found in the S1 Appendix. Before the game started, participants answered control questions. Afterwards, participants answered a short questionnaire.

The experiment was computerized using z-Tree [28] and conducted at a German university. Participants were students from the Faculty of Business, Economics and Law and were recruited using the data-base tool ORSEE [29]. The participation in the experiment was voluntary and the data were collected and analyzed anonymously. For the experiment no IRB was acquired as there is no internal review board at the university where the experiments were conducted. In accordance with the Declaration of Helsinki, all participants were requested to read an online consent form and agree with its terms (by clicking) before registering to take part in an experiment. Participants were guaranteed the anonymity of the data generated during the experiment. In each experiment subjects were informed that they participate in a study of decision making. For the recruitment procedure there was no specific inclusion criteria. In the experiment, participants were provided with an endowment of 350 ECUs (5.25) and their final payoffs were converted at the rate €0.015 per ECU. The average payment received was €13.49 (see Table 2 for a decomposition by treatment and role). An experimental session lasted approximately 90 minutes.

**Table 2:** Average payoffs by treatment and role. Numbers of participants in parentheses.

	Treatment *NR*	Treatment *SR*	Treatment *SRG*	All
Employers	€23.93 (36)	€21.47 (36)	€22.08 (18)	€22.57 (90)
Employees	€9.73 (72)	€8.93 (72)	€7.40 (36)	€8.94 (180)
All	€14.46 (108)	€13.11 (108)	€12.30 (54)	€13.49 (270)

We conducted ten sessions: four in each of the treatments *NR* and *SR*, and two for treatment *SRG*. We ran fewer sessions of *SRG*, as this treatment is very similar to *SR*. We had 27 participants in each session, which resulted in three matching groups per session. In total we had 270 participants, which generated 30 independent observations. In each treatment, half of the employees and about half of the employers were female. In treatments *NR* and *SRG*, exactly half of the employers were female. In treatment *SR*, 55.6% were. In treatment *SRG*, the gender composition of employee groups was fully balanced. In *SRG* sessions, there was one group of two male workers, one group of two female workers and one group of a male and a female worker in each matching group and period. In treatments *NR* and *SR*, in contrast, there were unequal and time-varying numbers of mixed, all-male and all-female groups (57.9% mixed groups in *NR*, 60.4% mixed groups in *SR*). Full gender group composition (i.e. including the employer’s gender) was not exactly balanced in any treatment. When recruiting subjects, we orientated on the sample size of other studies on multi-employee gift exchange games. For instance, [20] apply two treatments with a total of 144 subjects.

## Hypotheses

In this section, we present our hypotheses on gender differences. We study a reversed multi-employee gift-exchange setting, similar to [20]. In this framework, the two employees of a team simultaneously move first and decide on their contributions of effort. We modified this scenario to a setting, characterized by uncertainty on the contributed effort levels, as the employers cannot trace back the individual effort levels of the employees. The employer receives information on the joint effort contribution and decides on the remuneration for each of the employees. Thus, this framework is not only highly uncertain, but also it builds on trust. That is, employees contributing effort have to trust that this is reciprocated by the employer. Moreover, this situation resembles a risky situation for the first movers. This holds for all of our three treatments, since they build on the same fundamental framework. Experimental economics documented that women behave more risk averse [30] and trust less than men [4, 14, 15]. Therefore, we overall expect that female employees exert less effort than men. Moreover, there is evidence that women are more ‘betrayal averse’ than men in trusting contexts [31]. In the *SR* and *SRG* treatments, female employees can not only be exploited by an employer who pays a low wage, but also by their co-worker who may overstate the effort in the reporting decisions. Therefore, we expect that female employees trust even less in the the *SR* and *SRG* treatments. This derives the first hypothesis.

**Hypothesis 1: Efforts**
(a) Female employees contribute lower effort levels than male employees.
(b) The difference is more pronounced in SR and SRG

In our *SR* and *SRG* treatments employees have to report their contributions to the employer. The employer can only observe joint contributions of the team, but he/she cannot differentiate between the individual contribution levels. Thus, employees can misreport the contributions. This is an import feature of our self-reporting set-up, since one stereotypical hypothesis is that women behave more truthfully and men are more inclined to overstate their contributions. Viewing reporting as a matter of truth-telling, we can focus on the evidence of the experimental literature on dishonest behavior. The majority of studies finds that men behave more dishonestly than women in the context of black lies, i.e., lies that benefit the liar [11–13]. In the context of white lies, i.e., when lying benefits another person, the findings are reversed [12]. However, if employees misreport in the self-reporting setting, they cannot benefit another person. Thus, in our setting, inflating the reported contribution level is more or less characterized by a black lie. Therefore, we derive the next hypothesis.

**Hypothesis 2: Self-reports**
Female employees are less likely to overstate their effort levels than men.

Building on Hypothesis 1, we derive a hypothesis on gender-wage gaps in our treatments. Regarding the impact of effort, Hypothesis 1 states that women contribute less effort than men, which is more pronounced in the treatments *SR* and *SRG*. Generally, this should lower women’s wages in all-female groups, as the aggregate effort contribution is lower than for all-male groups. This should induce a gender-wage gap in *NR*, which is more pronounced in *SR* and *SRG*, since Hypothesis 1 expects a higher effort difference between female and male effort. Regarding the effects of gender observability, we expect that the gender-wage gap is amplified in *SRG* for several reasons. Gender observability may activate gender stereotypes, gender identity sentiments, and gender-contingent choices, including the possibility to discriminate. Furthermore, it may reduce the feeling of anonymity and affect learning by providing a salient piece of information about other participants. Therefore, it is possible that female employees may behave less competitive when realizing that co-workers are men [16]. If gender stereotypes play a role, this may also affect employers’ behavior, leading to discriminatory behavior against female employees. It may be that employers pay lower wages to female employees [6, 7]. This is also supported by evidence in Ultimatum games, where female responders receive lower offers than men. This holds for both genders in the role of proposers [32]. [33] highlight that women may especially be discriminated by other women. If female employees learn that they are discriminated against, it is possible that they anticipate this [10] and lower their contributions in the course of the game. Altogether we expect that employers reward higher effort by higher wages. Thus, women should receive lower wages, which leads to gender-wage gaps in all treatments. We expect that it is more pronounced in *SR* and most pronounced in *SRG*.

Regarding the effects of self-reports there are several possibilities. One possibility is that self-reporting makes no difference at all because employers disregard reports completely, considering that these reports have no direct monetary cost for employees, are unverifiable and in that sense constitute ‘cheap talk.’ However, if employers use the reported contributions as signals, self-reports should further amplify the wage gap. The reason is that overstating effort may help men capturing some of the co-worker’s wage share. It is unlikely that employers punish overstatements that are too large, since it is hard to figure out who has lied. We summarize our expectations on gender-wage gaps in Hypothesis 3.

**Hypothesis 3: Wages**
(a) Men receive higher wage payments than women, which yields a gender-wage gap in NR.
(b) The gender-wage gap is more pronounced in SR than in NR.
(c) In SRG, the gender-wage gap is most pronounced.

## Results

In this section we report our results on gender differences. In what follows, we use ‘contributions’ and ‘efforts’ interchangeably. In the present context, both terms refer to an action that is individually costly while beneficial at a group level. We first consider efforts, self-reports, and wages separately, showing graphical and statistical evidence of differences by treatment and gender. We always report results of two-sided non-parametric tests (if not otherwise stated). We use the following abbreviations: Wilcoxon signed-rank test (*WSR*), Mann-Whitney test (*MWU*). We also report the parametric results of an OLS regression on effort, self-reports, and wages. The regression table (Table 3) is presented after we report wages. The subsection thereafter analyzes employer choices, which determine employees’ incentives.

**Table 3:** OLS results for treatment and gender differences.

DV:	(1)	(2)	(3)	(4)
	Effort	Report	$\frac{\text{Report}}{\text{Effort}}$	Wage
*NR*	0.39			3.39
	(0.51)			(3.48)
*SRG*	-1.02*	-0.88	0.42	-6.09
	(0.53)	(0.76)	(0.43)	(3.80)
(*NR* − *SRG*)	1.41**			9.48**
	(0.60)			(3.86)
Female	-1.53***	-0.14	1.44***	-4.61***
	(0.29)	(0.40)	(0.45)	(1.41)
*NR* × female	0.63			0.45
	(0.47)			(2.13)
*SRG* × female	1.28**	0.12	-1.21*	2.76
	(0.59)	(0.51)	(0.66)	(2.06)
(*NR* − *SRG*) x Female	-0.64			-2.31
	(0.63)			(2.20)
Constant	4.47***	6.90***	2.39***	16.86***
	(0.29)	(0.39)	(0.15)	(2.41)
*N*	4320	2592	2592	4320
*R*^2^	0.065	0.022	0.052	0.066

* p<0.1,

** p<0.05,

*** p<0.01.

Standard errors clustered at the matching group level in parentheses.

Treatment *SR* and men constitute the omitted reference categories.

### Employee choices and outcomes

#### Gender differences in effort provision

**Fig 1:**
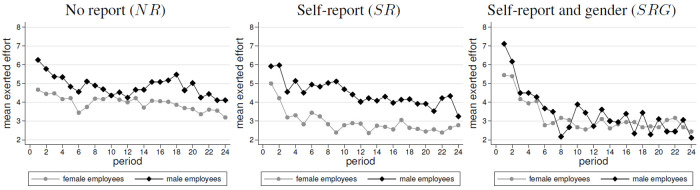
Average exerted efforts by treatment and gender (order: *NR*, *SR*, *SRG*).

Recall from the experimental design section that the amount of effort provision is tantamount to the efficiency of the production interaction. Fig 1 shows average effort over time in the three treatments, separately for men and women. In all treatments, average effort declined significantly over time (Spearman rank-correlation tests, henceforth ‘*SRC* test’: *ρ* = −0.210, *p* < 0.001 for treatment *NR*; *ρ* = −0.308, *p* < 0.001 treatment *SR*; *ρ* = −0.457, *p* < 0.001 treatment *SRG*).

In the absence of self-reports, we find in *NR* that women contributed somewhat less effort than men (3.96 vs. 4.86). In *SR*, the gender gap in effort is more pronounced in the presence of self-reports (women: 2.94; men: 4.47). Hence, female employees contribute significantly less effort than men, in both treatments. This is confirmed by *WSR* tests. (*NR*: one-sided test *p* = 0.059; *SR*: two-sided test *p* = 0.003) and by OLS regressions in Table 3 (*NR*: *p* < 0.05; *SR*: *p* < 0.01). To test for gender differences within one matching group, we apply Wilcoxon matched-pairs tests, since we are interested in statistical differences of two outcomes (i.e., the means of men and women), which stem from the same independent observation, i.e., our match group. This is appropriate in our matching design, since we have mixed gender compositions in our match groups, where men and women repeatedly interact. We follow the approach of [34] who also apply Wilcoxon matched-pairs tests to test gender differences of men and women in the same match group.

We find that the gender gap is more pronounced, since female effort is more sensitive to the availability of self-reports, which leads to a lower female effort (*MWU*, *p* = 0.043; OLS, *p* = 0.052, see Table 3). By contrast, we do not find this effect for male employees. Their contributions are always higher than females’ contributions and they do not differ between the availability of self-reports (*MWU*, *p* = 0.507; OLS, *p* = 0.445, see Table 3). Interestingly, the gender gap in effort vanishes in *SRG*, since male employees decrease over time their effort contributions. As a consequence, we find in *SRG* similar effort contributions, which do not significantly differ between both genders (men: 3.45, women: 3.19, *WSR*, *p* = 0.528, OLS, *p* = 0.621, see Table 3). To summarize, we find support for Hypothesis 1a in treatments *NR* and *SR*, but not in *SRG*. The difference in effort is more pronounced in *SR* than in *NR*. However, we do not find that *SRG* leads to the highest difference. Therefore, we only partially support Hypothesis 1b.

**Result 1 (Efforts)**
(i) Women provided a lower level of effort than men in NR. This is especially pronounced in SR.
(ii) In SRG, the average effort levels of men and women were not significantly different.

#### Self-reported efforts of women and men

**Fig 2:**
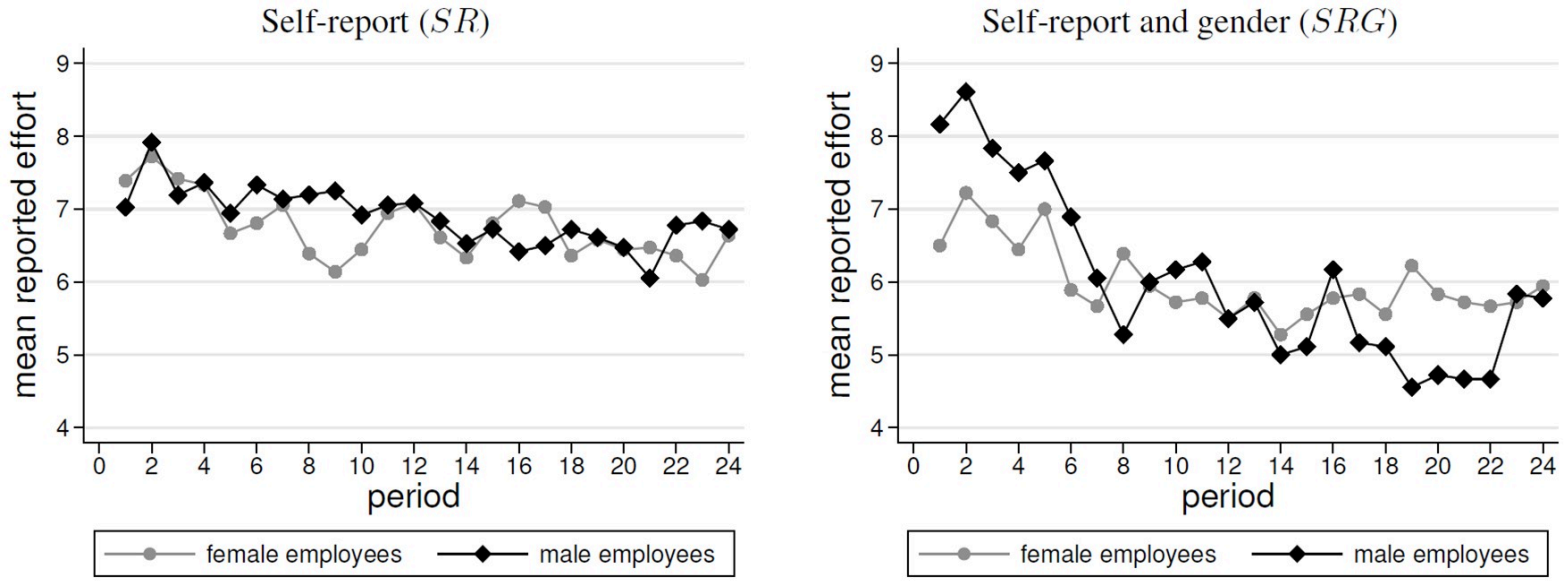
Average reported efforts by treatment and gender (order: *SR*, *SRG*).

**Fig 3:**
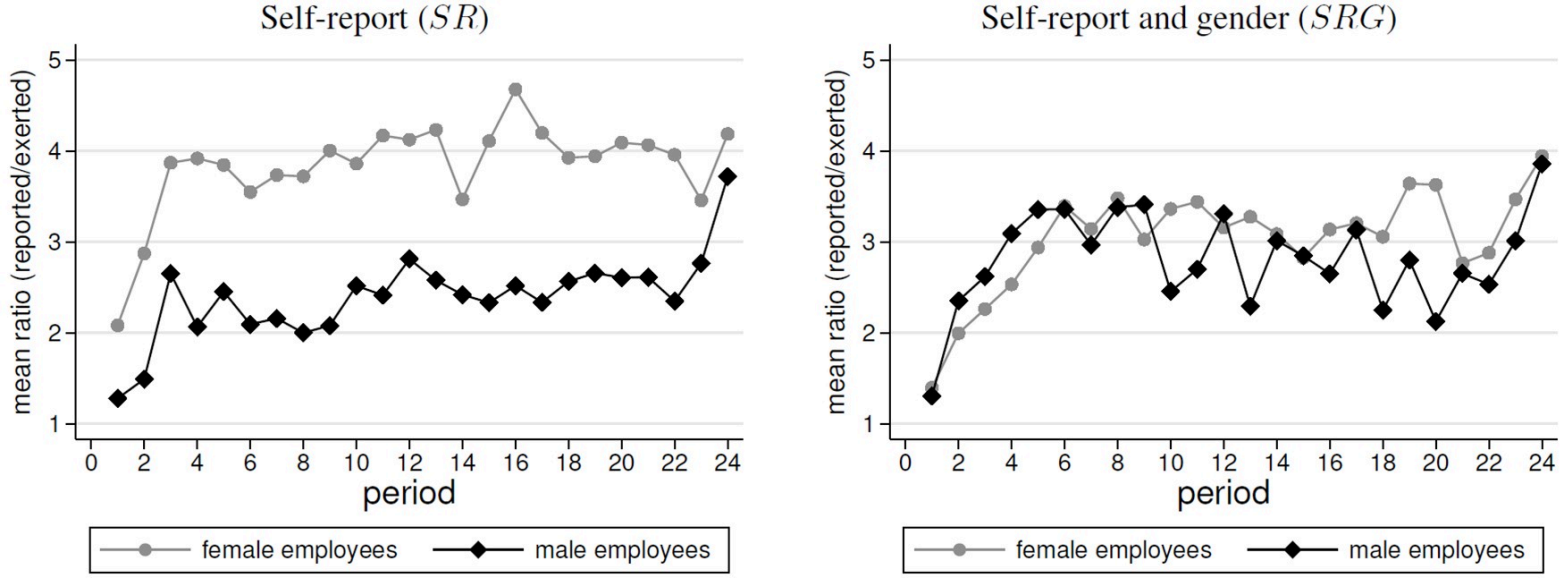
Average ratio of reported to exerted efforts by treatment and gender. The displayed average ratio of reported to exerted efforts does not equal the ratio of average reports (Fig 2) to average efforts (Fig 1).

The previous subsection revealed that a female-male effort gap emerged when gender was unknown. The gap was largest when efforts were self-reported. In this subsection, we examine how this relates to the effort levels that were reported. Fig 2 overviews the average reported effort levels, whereas Fig 3 displays the average differences in the individual ratios of reported to exerted efforts.

In both treatments with self-reports, the level of reported efforts declined significantly over time (*ρ* = −0.168, *p* < 0.01 in *SR*; *ρ* = −0.391, *p* < 0.001 in *SRG*; *SRC* tests). We find that in both treatments, the levels of reported efforts of men and women were not significantly different (in both treatments: all *p* − *values* > 0.5 for *WSR* and OLS, see Table 3). However, this does not mean that men and women do not differ in the level of overstatement. In fact, women exerted less effort in *SR*. As a consequence, the similar levels of reported effort imply that women overstated their efforts more. This is documented by Fig 3. It can be seen that in *NR* and *SR*, the gender difference in effort implies a larger degree of overstating for women (by factor 3.83) than for men (by factor 2.39) (*WSR*, *p* = 0.015, OLS, *p* < 0.01, see Table 3). Focusing on the percentage of truthful vs. untruthful reports, we find that women reported truthfully in only 10% of the cases, while men behave so in 22% of the cases. By contrast, in Fig 3 we find no gender difference when focusing on *SRG* (*WSR*, *p* = 0.753; OLS, *p* = 0.667, see Table 3). Similarly, we find that men and women reported truthfully about 25% of the time.

**Fig 4:**
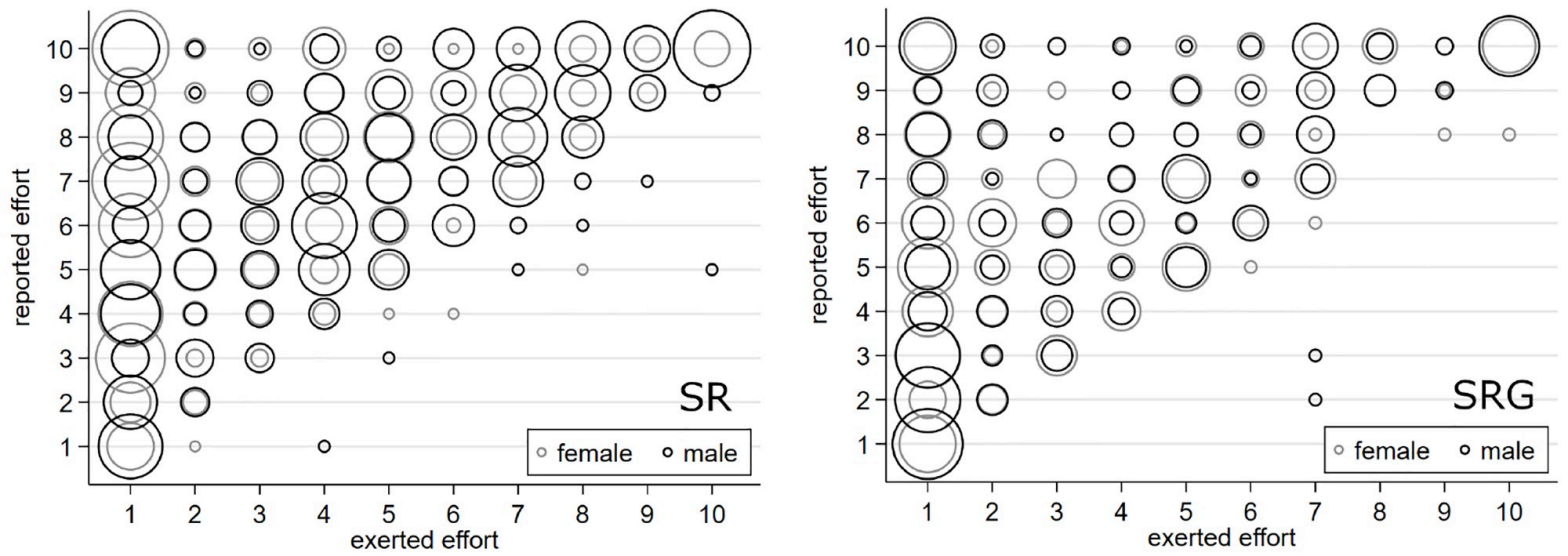
Frequency of certain combinations of exerted and reported effort levels.

To get more insights about the reporting behavior of women and men, we present two bubble plots, which focus on the frequency of certain combinations of exerted and reported effort levels (Fig 4). The figure focuses on the *SR* treatment (left panel) and on the *SRG* treatment (right panel). Men are presented by black circles, whereas, women are presented by gray circles. Larger bubbles indicate that this scenario occurred more often.

In the *SR* treatment, both gender most often exerted effort levels of 1 and reported various levels higher than 1. This behavior is especially pronounced for female employees, which can be seen by the larger radius of the gray circles. Turning to the *SRG* treatment, we find that subjects’ behavior is more concentrated to the cases, where an effort of 1 is exerted and higher levels are reported. The right panel shows that this holds for both genders.

In summary, this section shows that we find no support for Hypothesis 2. That is, women do not behave less dishonestly than men. We find similar reported absolute effort levels in *SR* and *SRG*. Turning to the data on overstated effort, we even find evidence for the opposite in *SR*. Whereas, no difference can be found in *SRG*. Thus, our results differ from the findings of the experimental literature on gender differences in honesty. An explanation may be that the employees coordinate with respect to the self-report, which is observable, and this implies a higher overreporting by women, who exert less effort. However, the latter is not observable for the employees.

**Result 2 (Self-reports)**
(i) On average, participants overstated their effort contributions.
(ii) Women reported similar effort levels as men.
(iii) In SR, women reported less truthfully and overstated their efforts to a higher degree than men.

#### Wages

**Fig 5:**
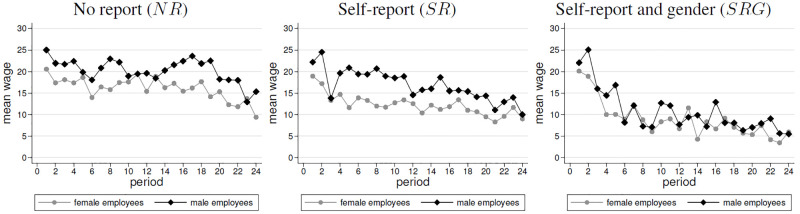
Average wages by treatment and gender.

Next, we analyze employees’ wages to test Hypothesis 3. Fig 5 shows the evolution of average wages by treatment and gender.

In all treatments, there was a general downward trend in wages (*SRC* tests: *ρ* = −0.178, *p* < 0.01 for *NR*; *ρ* = −0.247, *p* < 0.001 for *SR*; *ρ* = −0.383, *p* < 0.001 for *SRG*). We observe a gender-wage gape in the *NR* and in the *SR* treatment. That is, female employees earned significantly less than male employees without self-reports (*WSR*, *p* = 0.060) and in the case of the *SR* treatment (*WSR*, *p* = 0.019). This supports hypotheses 3a and 3b. By contrast, in *SRG*, average wages were not significantly different between men and women (*p* = 0.463, *WSR*). This rejects Hypothesis 3c.

**Result 3 (Wages)**
(i) With and without self-reports, a wage gap occurs, i.e., female employees receive lower wages than male employees.
(ii) The gender-wage gap does not occur in SRG.

### Employers’ reactions

#### Wage-effort relation: The role of co-workers’ gender

In this section we focus on the reaction of employers on employees’ choices to learn why we observe a gender-wage gap in our data. We start with the wage-effort relation and analyze whether paid wages depend on co-workers’ gender. This may help to learn what drives the gender-wage gap we observe.

**Fig 6:**
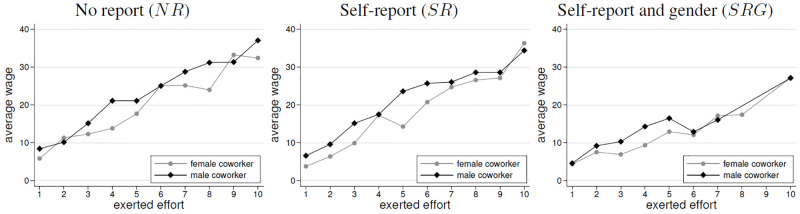
Wage-effort relations by co-worker’s gender. Only data points with at least ten underlying observations are shown.

We start with the role of employees’ effort choices for their received wages. That is, we analyze how the individual wage-effort relation varies with the co-worker’s gender. Fig 6 plots this relation separately for each treatment.

As expected, we observe that wages are increasing in the level of contributed effort in all treatments. This confirms the gift-exchange relationship in our setting [17, 18, 20]. Given the tendency of lower female efforts in all treatments and the fact that employers could not perfectly attribute efforts, having a female co-worker yields a lower wage for a given effort. The diagram suggests that this holds in *SR* and *NR* as well. Nevertheless, in fixed-effects regressions we only find in *SR* that this association is significant. This is consistent with the largest gender effort gap, which is pronounced in the self-report treatment. The regressions are presented in Table 4 and focus on the wage-effort relation conditioned on the co-worker gender. Again, we omit the *SR* treatment. The models focus on the paid wage, conditional on the exerted effort. To identify a co-worker’s gender, we include a dummy (*female co-worker*). We also focus on treatment interactions with effort and with the gender of the co-worker.

**Table 4:** Wage-effort regressions by co-worker gender (employer fixed effects).

DV: Wage	(1)	(2)	(3)	(4)
	*NR*	*SR*	*SRG*	all
Effort	2.27***	2.56***	2.14***	2.56***
	(0.19)	(0.19)	(0.48)	(0.18)
Female co-worker	-1.49	-1.59**	-1.17	-1.59**
	(1.06)	(0.65)	(1.13)	(0.63)
*NR* × effort				-0.29
				(0.26)
*SRG* × effort				-0.42
				(0.49)
*NR* × female co-worker				0.10
				(1.21)
*SRG* × female co-worker				0.42
				(1.23)
Constant	8.92***	5.88***	3.32	6.58**
	(1.06)	(0.75)	(1.31)	(0.56)
*N*	1728	1728	864	4320
*R*^2^	0.61	0.71	0.55	0.65

* p<0.1,

** p<0.05,

*** p<0.01.

Male co-workers (and in column (4), treatment *SR*) constitute

the omitted reference categories.

All models include employer fixed effects.

It can be seen that *effort* is always significant, which confirms the gift-exchange relationship, observed in the diagrams. In model (2), the significant negative coefficient of *female co-worker* highlights that a female co-worker particularly lowers wages in the treatment with self-reports. This also suggests, employees in all-female groups receive the lowest wages, which apparently determines the gender-wage gap.

**Result 4 (Co-worker gender)**

In SR, employees receive significantly lower wages when their co-workers is female.

The finding shows that at least parts of the gender-wage gap are induced by the behavior of female employees. Fig 6 suggests that this negative effect of female co-workers holds in *NR* and in *SR*, but not in *SRG*. Moreover, we find that the difference of exerted efforts between male and female employees is largest in *NR* and *SR*. Whereas, in *SRG* men contribute a similarly low effort as women. If self-reports would be the driving force for the gender-wage gap, we should not observe a negative effect of a female co-worker in *NR*. Moreover, we should observe a negative effect in *SRG*, which is not the case. In the next section we deeper explore the possible effects of self-reports on the reaction of employers’ wage-setting behavior.

#### Self-reports and wage setting

In this section, we briefly analyze the role of self-reports on the received wages. The analysis of self-reported effort levels showed in *SR* and *SRG* that no gender differences exist in the absolute reported effort levels. However, we found that women clearly overstated their efforts more pronouncedly than men. Hence, if employers punish overreports by paying lower wages to employees who clearly lied, it is possible that this drives the wage gap we observe, since female employees overreport to a higher extent than men. There is only one case, where it is clear that one of the two employees must be lying (i.e., the case of an effort sum of two and at least one employee reports an effort level of greater than one). Therefore, it is possible that punishment behavior of the employers may result in lower wage payments to female employees, whenever they overreport in the case when the reported effort sum of both employees is two. To deeper explore this, we focus on employers’ reactions in the aggregate data of self-reports and resulting wage payments.

**Fig 7:**
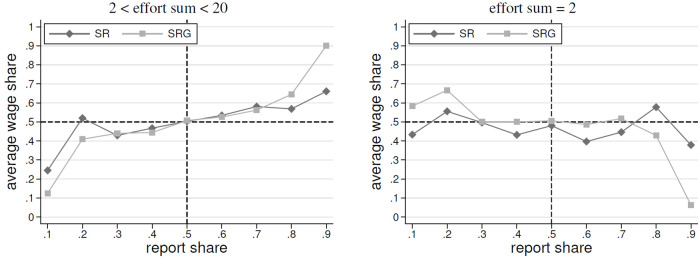
Average wage shares by share in reports.

Fig 7 documents the impact of the *report share* on the average wage share. The report share is the share of the sum of reported self-reports, which is reported by an employee. In the left panel we present all cases where the sum of effort is between 2 and 20, i.e., in these cases the effort sum does not reveal individual effort. Whereas, the right panel focuses on all cases where the effort sum is 2, i.e., reporting a share above 50 percent reveals that a person overreports.

The left diagram shows that an employee’s wage share is indeed related to her share in the reports. Thus, it rather paid off to report more than the co-worker. The right panel highlights that when effort is obviously minimal, higher reported shares appear to have virtually no effects in *SR*. That is, employers do not punish by lowering wage payments. In *SRG* there is evidence for little punishment of very high overreportings. Thus, we conclude that lower wages of female employees cannot be related to the fact that they overreport more than their co-workers. This adds further evidence that women’s self-reporting behavior does not drive the gender-wage gap.

**Result 5 (Wage Reactions to Self-Reports)**
Employers do not punish overreporting in SR. Thus, the gender-wage gap cannot be explained by female employees’ overreporting behavior.

In the next subsection, we concentrate on the *SRG* treatment and analyze whether female employees are discriminated by employers.

#### Evidence for discrimination

In treatment *SRG*, employers observe their employees’ genders and therefore may discriminate them by gender. The regression results of Table 5 provide evidence that female employers discriminate against female employees: controlling for effort sums and reported efforts, the coefficients of the interaction between female employers and employees are significantly negative at the ten percent level. There is some indication that such discrimination was related to stronger punishment of female overreporting: for minimal effort sums, the relation between report and wage has a significantly more negative slope for a female employee in OLS (at *p* < 0.1), random employer effects (at *p* < 0.05). The discrimination finding is consistent with a finding from dictator games, in which women gave significantly less to other women [33].

**Table 5:** Gender discrimination regressions in treatment *SRG*.

DV: Wage	(1)	(2)	(3)	(4)
	OLS	RE	OLS	RE
Female employee	0.36	0.67	0.20	0.84
	(0.69)	(1.04)	(0.75)	(0.94)
Female employer	2.99	2.84	3.00	2.85
	(4.02)	(4.01)	(3.67)	(4.18)
Female employee × female employer	-3.40	-3.10*	-3.27*	-3.28*
	(1.76)	(1.65)	(1.59)	(1.69)
Effort sum	1.74***	1.77***	1.44***	1.94***
	(0.37)	(0.38)	(0.35)	(0.36)
Report			0.76**	0.02
			(0.29)	(0.11)
Co-worker report			0.14	-0.60***
			(0.09)	(0.19)
Constant	-2.50	-2.86	-5.92	-0.51
	(1.74)	(1.83)	(3.33)	(1.46)
*N*	864	864	864	864

* p<0.1,

** p<0.05,

*** p<0.01.

Standard errors clustered at the matching group level in parentheses.

Male employer-male employee constitutes the omitted reference category.

Columns (2) and (4) include random employer effects.

**Result 6 (Female-to-female discrimination)**
Female employers appear to discriminate against female employees when gender information is available.

## Discussion

We studied gender differences in a reversed gift-exchange game with multi-employees, where effort was not observable. In this respect, we tested the effects of self-reports on employee behavior. The data show clear evidence for a gender-wage gap, i.e., female employees receive lower wages than men in the treatment with self-reports (*SR*). A closer look at the data showed that apparently a main driver is that female employees always exerted a very low level of effort in each of the three treatments we studied. We find evidence for significant gender-wage gaps in the treatments with self-reports (*SR*) and without self-reports (*NR*), since men always exert higher effort levels in theses treatments. Therefore, purely male groups are better paid than purely female groups (while in mixed groups male and females are on average paid the same). The wage difference vanishes in the *SRG* treatment, where men converge to a similar low effort level as women.

Our analysis on employers’ reactions revealed that there exists a very strong wage-effort relation in our data. That is, employers pay high wages to the employees, whenever the two employees exert a high total-effort sum. This suggests, that combinations where employees are matched with female employees should systematically lower the received wage payments. Therefore, we focused in a next step in regressions on the impact of a female co-worker. The regressions indeed confirm the idea, that the gender-wage gap is driven by worker groups composed of females. In line with our main findings, the regressions highlighted that the gender effects are especially pronounced in the *SR* treatment. Does this mean that self-reports lead to a behavior of employees, which induces gender-wage gaps? Several arguments speak against that. First, the wage gap is not exclusive to our *SR* treatment, i.e., we also find it in the *NR* treatment (without self-reports), but not in *SRG* (where self-reports are in the place). Second, if self-reports would drive the gender effect, we should observe that females’ self-reporting behavior systematically differs from the one of men and that employers react to this. This is not the case for the observable self-report levels. Although, women overstate their effort to a higher extent than men, we observe that both genders report the same absolute effort levels. Note that our design leaves only little room for employers to clearly identify employees who lie (this is only possible for the case of the minimum effort sum of two). However, the data show that employers do not punish employees in the case when they overstate their efforts. It can be seen that they even reward employees who report more than their co-worker. Based on this, we cannot deduce that female employees who report similar effort levels as men, earn less because of their reporting behavior.

Overall, the gender-wage gap obviously occurs as a consequence of the generally low effort of female employees in this setting. In this respect, the behavior confirms the evidence of trust games, which shows that female first movers trust less than male first movers. The remaining question is: why do we observe a treatment effect for the gender-wage gap when we introduce self-reports? Again, the explanation is the effort level of female employees, which decreases in this case. A reason may be that in this environment women face even more uncertainty than in the case without self-reports. That is, they have to form a belief on the (dis)honesty of the co-worker and of the reaction of the employer when observing both, her own report and the report of the co-worker. Probably this more uncertain work environment further lowers trust of female employees. Moreover, women may feel exploited, if their effort is not rewarded by the employers. This feeling may be amplified in the presence of self-reports, since women may also feel betrayed by their co-worker, if he/she cheats by reporting a wrong effort. As a consequence, female employees distrust more and exert less effort in the presence of a self-reporting institution, which is based on cheap talk. Finally, we discuss why we observe that the gender-wage gap shrinks when we reveal information about the gender of the players. To understand this, we again have to focus on the effort levels. It can be seen that the disappearance of the wage gap is mainly induced by men who clearly lower effort in *SRG* (3.45) as compared to *SR* (4.47) and *NR* (4.86). A closer look at the time dynamics shows that male employees converge to the low effort levels of female co-workers. We speculate that in *SRG* they imitate the behavior of females in the course of the game. This can be observed in Fig 3, which documents that the ratio of self-reported effort and exerted effort increases over time. Here, we observe for both genders very similar trends in the overreporting behavior. Overall, in *SRG* both genders seem to imitate their behavior, which leads to similar low efforts and similar low wages. As a consequence, no gender-wage gap occurs.

## Conclusion

In this paper, we documented that gender effort and wage gaps can emerge and persist in multi-employee gift exchange environments with unobserved individual contributions. More specifically, we found that in *NR* and *SR*, women contributed less than men, in particular when contributions were self-reported. At the same time, they reported similar levels of contributions, implying a higher degree of overreporting. In mixed gender encounters, these choices amounted to inadvertently taking advantage of higher male efforts. But overall, lower average contributions from females resulted in lower female wages. In contrast, in *SRG*, initial differences in behavior leveled out in the course of the interaction.

The results suggest that parts of wage gaps may be related to different behavior in environments characterized by incomplete contracts and imperfect information. The results further show that this depends on details of the informational context. The study provides an example of gender-wage gaps that stem from differences in contributions and do not translate to gaps in economic rents, since women also incurred lower effort costs. Needless to say that the experiment presents a very stylized environment. Therefore, care must be taken in drawing direct parallels to real labor markets [35]. The experiment draws attention to features that may be important in real labor markets, namely the consequences of self-reporting institutions, the interaction of employee heterogeneity, and the observability of contributions and characteristics. The fact that in the highly uncertain *SR* environment the largest treatment differences occurred for women, is in line with the argument that women tend to react more strongly to social cues [4, 36]. Interestingly, we did not find gender differences “(…) to be more forcefully expressed in environments in which the gender of the other subjects is known (…)”, as posited by [37, p. 34]; quite to the contrary, gender differences were largest when gender was not observed.

An important question for future research will be to find out precisely what factors caused women to contribute less in the treatments without gender observability (*NR*, *SR*). Among the possible explanations is a lower level of trust, which may be the result of the uncertain environment. It is emphasized in the situation where women additionally have to make a (cheap-talk) report, not knowing the (dis)honesty of their colleagues. In this setting, women may trust less and exert less effort, as they anticipate the feeling of betrayal when receiving low wages from the employer, or when being cheated by the co-workers. Other reasons may be weaker preferences for efficiency and a higher concern for equality to avoid falling behind [26]. Such ‘preferences’ might also be intertwined with a history of employers actually paying lower wages for female efforts [38]. These factors are particularly suited to explain initial gender gaps in effort. In light of the stereotypical perception of more benign female social preferences and the feedback given, the persistence of these effort gaps is more surprising. It may also be worthwhile to further investigate the causal channels behind the disappearance of gender gaps with observable gender. While the first-period behavior in that treatment is suggestive of gender identity effects [39, 40], the convergence that occurred over time need not have had a gender-specific component. Finally, it could be interesting to study other, less restricted forms of communication, which a related literature recently suggested as effective at sustaining efficient outcomes [41]. In the absence of direct information on individual contributions, understanding the details of reporting and coordination mechanisms within groups may be crucial to understanding what makes diverse groups successful.

## Supporting information

S1 Appendix Instructions (translated from German). (PDF)
