# Supplementary material for: Gender differences in multi-employee gift exchange with self-reported contributions
Source: PLoS One. 2020 Sep 1;15(9):e0238236. doi: 10.1371/journal.pone.0238236 (PMC7462294; doi:10.1371/journal.pone.0238236)
Supplement: S1 Appendix — (PDF) [file pone.0238236.s001.pdf]

Instructions for the experiment

In this experiment, you will either take the role of an employer or an employee.[1] The role will be randomly assigned at the beginning of the experiment and will remain the same throughout.

The experiment will run for 24 rounds. In each round, groups of three will be formed, with each group consisting of one employer and two employees, employee 1 (EM1**, m/f**) and employee 2 (EM2**, m/f**). In each round, new groups of three will be formed randomly so that you will interact with different participants. At no point in time will you be informed about the identity of other participants. **You will, however, receive information about the gender of other participants (m/f).** You will not be able to track if you have already interacted with the same persons before. In what follows, you will receive detailed information about the stages of the experiment.

Each round consists of the following stages:

1.) Stage 1: Choice of Effort
2.) Stage 2: Report of Effort
3.) Stage *2***3**: Choice of Wages

See the following pages for more information.

[1] **The original instructions in German for treatment SRG explicitly mention the female and male variants of employer and employee in the first sentence. They then state that for ease of readability, employers and employees will subsequently be referred to as "employee (m/f)" and employer (m/f)" (instead of always repeating the male and female version of the words "employer" and "employee" in German).**

Instructions for the experiment

Stage 1: Choice of effort

In the first stage, employee 1 **(m/f)** and employee 2 **(m/f)** choose their respective effort level simultaneously and independently of one another.

There are ten effort levels from which you can choose. The lowest effort level is 1 and the highest is 10. Each unit of effort generates a revenue of 10 points for the employer **(m/f)**. In other words, if the amount of effort is 1, the employer **(m/f)** receives 10 points; if the amount of effort is 2, the employer receives 20 points and so forth. If the amount of effort is 10, the employer receives 100 points.

Throughout the whole experiment, the employer **(m/f)** cannot observe the effort provided by an individual employee, but only the sum of generated revenues by the two employees **(m/f)**. We will come back to this later.

The choice of effort is costly for an employee **(m/f)**: higher efforts are more costly. The costs for an employee **(m/f)** only depend on her own choice of effort. The effort level chosen by the co-worker **(m/f)** has no influence on these costs. The effort costs of an employee **(m/f)** are as follows:

Effort level:	1	2	3	4	5	6	7	8	9	10	
Cost:	0	1	2	4	6	8	10	13	16	20	points

An effort level of 1 has no cost for the employee **(m/f)**; an effort level of 2 costs one point and so forth. An effort level of 10 costs 20 points. The cost table is the same for all employees **(m/f)** and all rounds.

Instructions for the experiment

Illustration: Screen Shot Choice of Effort

Here you can see the screen on which an employee **(m/f)** makes his/her choice of effort.

[TREATMENTS NR/SR:]

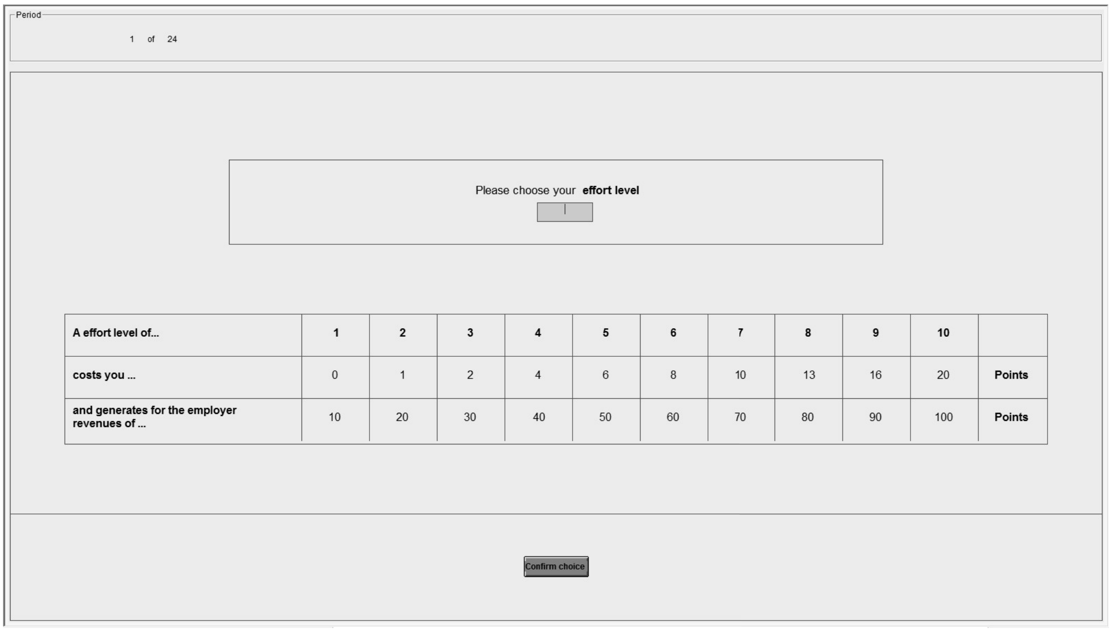

A effort level of...	1	2	3	4	5	6	7	8	9	10	
costs you ...	0	1	2	4	6	8	10	13	16	20	Points
and generates for the employer revenues of ...	10	20	30	40	50	60	70	80	90	100	Points

[TREATMENT SRG:]

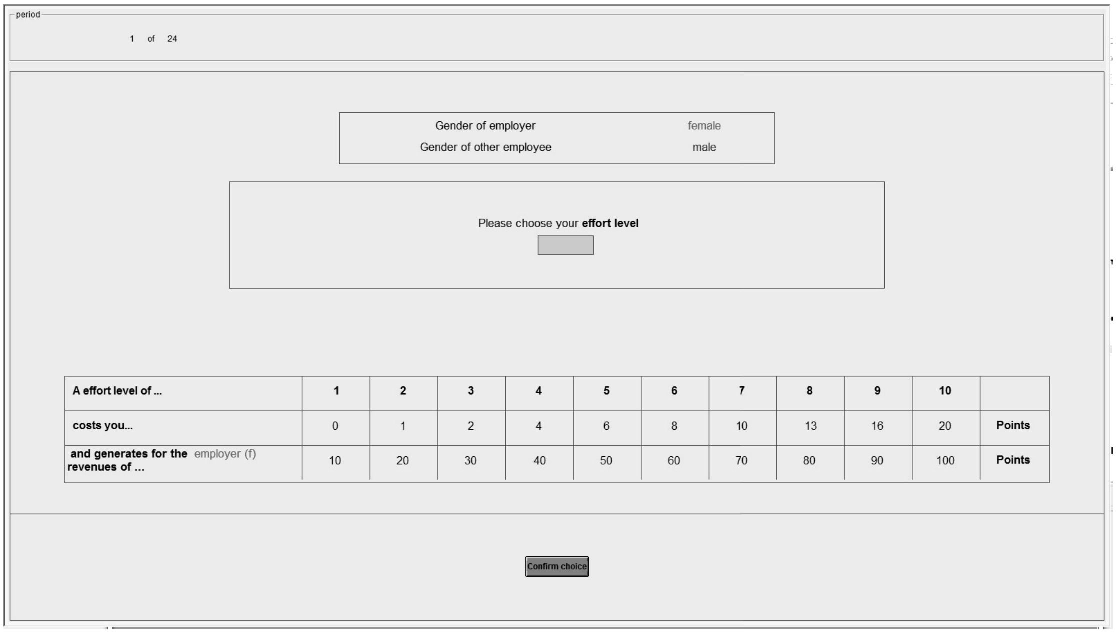

A effort level of ...	1	2	3	4	5	6	7	8	9	10	
costs you...	0	1	2	4	6	8	10	13	16	20	Points
and generates for the employer (f) revenues of ...	10	20	30	40	50	60	70	80	90	100	Points

Instructions for the experiment

- In the lower section, you find the cost table and the associated revenues generated for the employer by the respective effort level.

- In the upper section, **you find information about the gender of the employer (m/f) and the other employee (m/f).** You find an input field wherein employees **(m/f)** make their effort level choice.

- Employees **(m/f)** choose their effort level by writing the desired effort level number in the blue input field. They then confirm that choice by clicking on the red button.

As mentioned previously, the employer (m/f) cannot observe the effort chosen by an individual employee. **However, in stage 2, employees (m/f) have to make a report about their chosen effort level to the employer (m/f).**

Stage 2: Report of Effort

In the second stage employee 1 (m/f) and employee 2 (m/f) report their effort simultaneously and independently of one another to the employer (m/f).

- **Any effort level between 1 and 10 can be reported to the employer (m/f).**
- **An employee's (m/f) cost of effort only depends on the chosen effort in stage 1 and is not affected by reporting a different effort level in stage 2.**
- **Also the revenues generated by an employee (m/f) depend only on the stage 1 decision and are not affected by the reporting choice in stage 2.**

Illustration: Screen Shot Report of Effort

Here you can see the screen on which an employee (m/f) reports her effort level to the employer (m/f).

[TREATMENT SR:]

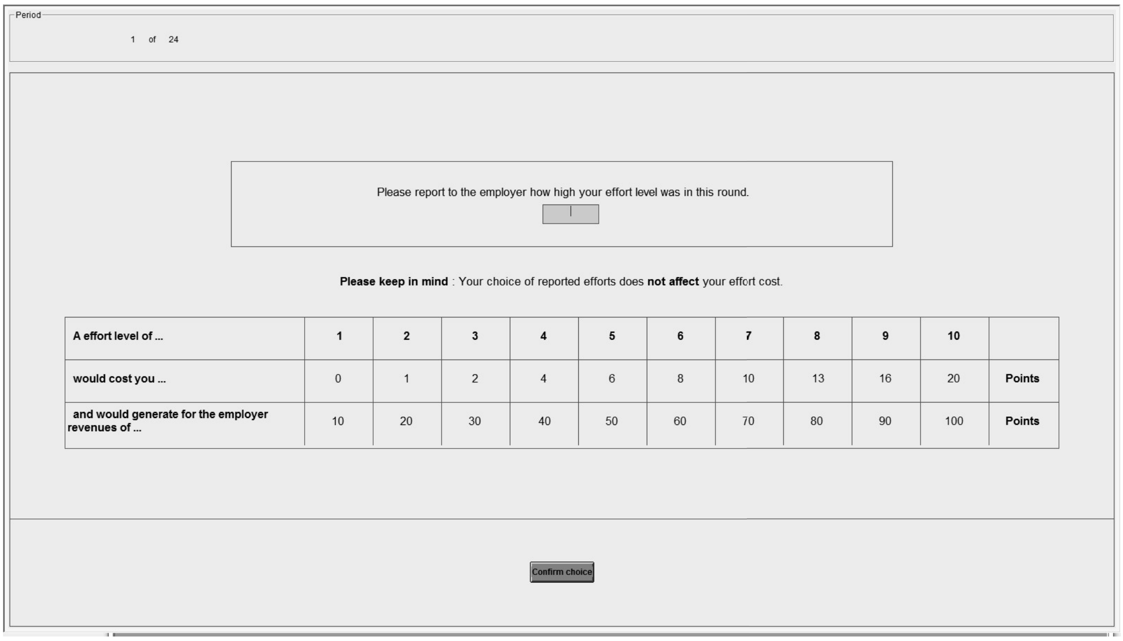

Period

1 of 24

Please report to the employer how high your effort level was in this round.

Please keep in mind : Your choice of reported efforts does **not affect** your effort cost.

A effort level of ...	1	2	3	4	5	6	7	8	9	10	
would cost you ...	0	1	2	4	6	8	10	13	16	20	Points
and would generate for the employer revenues of ...	10	20	30	40	50	60	70	80	90	100	Points

Confirm choice

Instructions for the experiment

[TREATMENT SRG:]

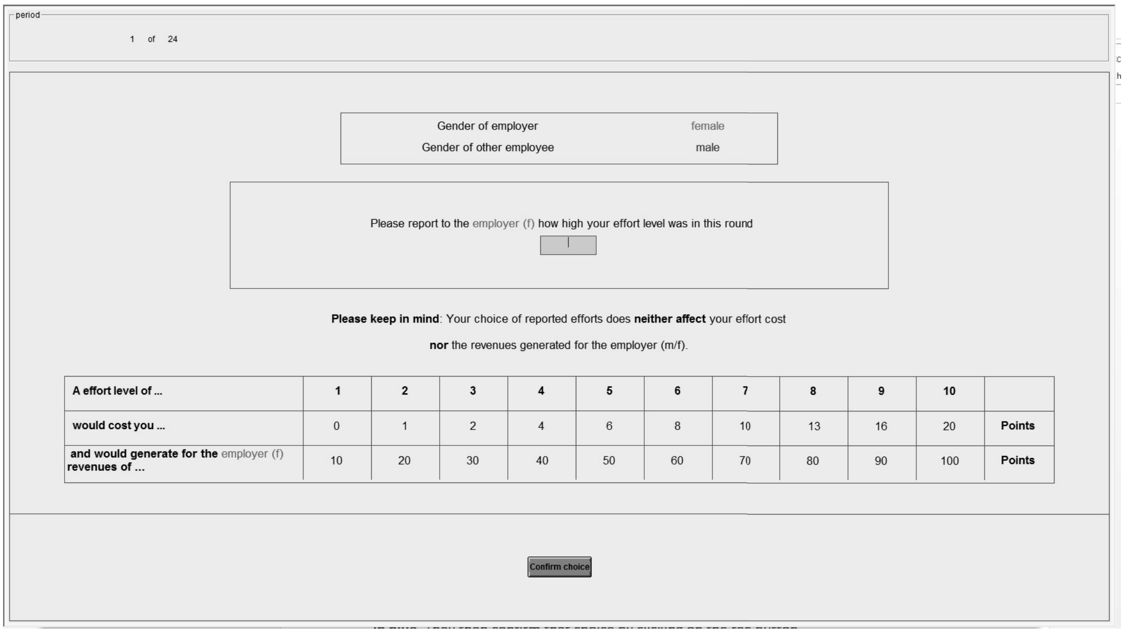

period

1 of 24

Gender of employer female

Gender of other employee male

Please report to the employer (f) how high your effort level was in this round

Please keep in mind: Your choice of reported efforts does **neither affect** your effort cost

nor the revenues generated for the employer (m/f).

**A effort level of ...**	**1**	**2**	**3**	**4**	**5**	**6**	**7**	**8**	**9**	**10**	
**would cost you ...**	0	1	2	4	6	8	10	13	16	20	**Points**
**and would generate for the** employer (f) **revenues of ...**	10	20	30	40	50	60	70	80	90	100	**Points**

Confirm choice

- **In the lower section, you find the cost table and the associated revenues that would be generated for the employer (m/f) by the respective effort level. Note: an employee's (m/f) effort cost is determined by the effort chosen in stage 1 and remains unaffected by a different choice in stage 2.**
- **In the upper section, you can find information about the gender of the employer (m/f) and the other employee (m/f). You also find an input field where employees (m/f) enter their choice of effort to report to the employer (m/f) in this round.**
- **Employees (m/f) report their effort level by writing the desired number in the input field in blue. They then confirm that choice by clicking on the red button.**

Please keep in mind:

- **The choice of reported effort in stage 2 does neither affect the employee's effort cost nor the revenues generated for the employer (m/f).**

- **The employer (m/f) will only informed about the reported effort levels of stage 2 and not about the actual chosen effort levels of stage 1. In addition, the employer (m/f) is informed about the sum of revenues generated by the two employees, i.e. total revenues.**

- *The employer will only be informed about the sum of revenues generated by the two employees, i.e. total revenues.*

- Summing up, the employer **<u>(m/f)</u>** receives the following information after stage *1***2**:

  - Total revenues=10*(effort level of EM1 + effort level of EM2)
  - **The reported effort levels of EM1 and EM2 from stage 2**

The employer **<u>(m/f)</u>** receives the above-mentioned information and subsequently determines the wages of both employees **<u>(m/f)</u>**.

Stage *2*3: Choice of Wages

In the *second***third** stage, the employer **(m/f)** is informed about:

1.) The total revenues that were generated by the sum of efforts of both employees **(m/f)** in this round.
2.) The reported efforts of EM1 (m/f) and EM2 (m/f) in this round.

The employer **(m/f)** then decides about the wages w1 and w2 for EM1 and EM2. The wages for individual employees **(m/f)** can either be the same or different. The wage for an individual employee **(m/f)** cannot be lower than 0 points and not be higher than 100 points.

Illustration: Screen Shot Choice of Wages

Here you can see the screen on which an employer **(m/f)** chooses the wages w1 and w2. This screen is only visible for employers **(m/f)**.

[TREATMENT NR:]

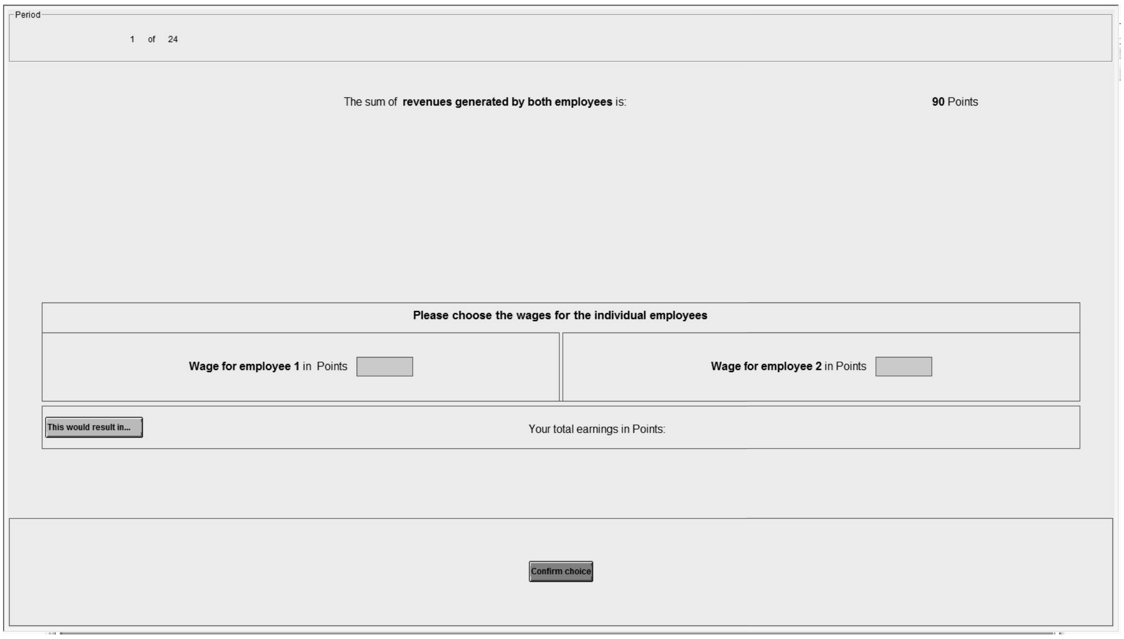

Instructions for the experiment

[TREATMENT SR:]

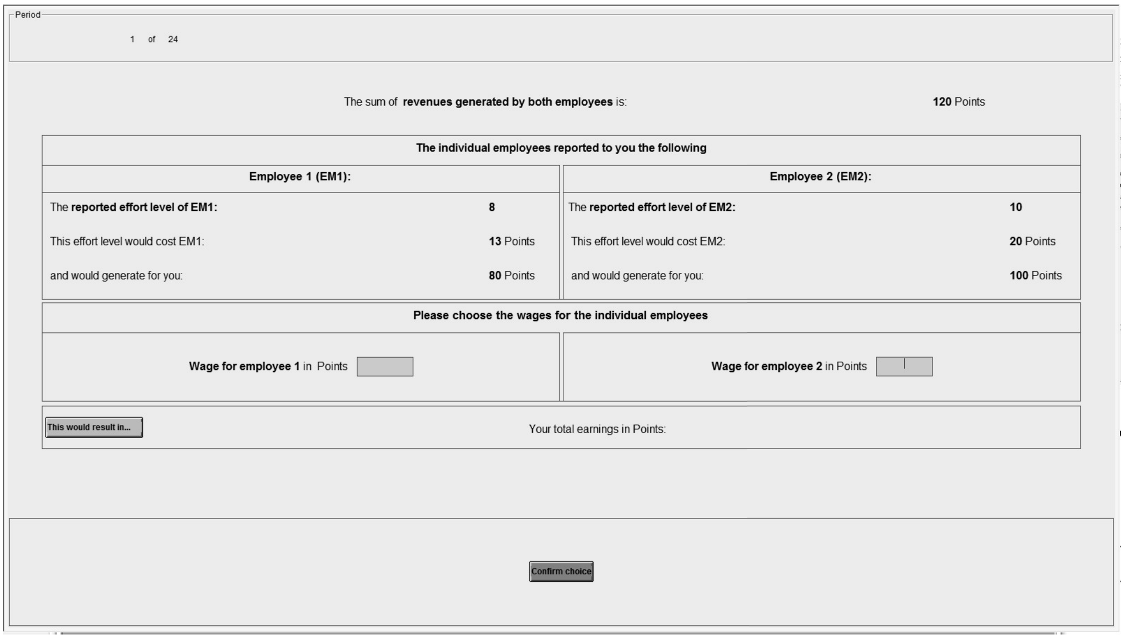

[TREATMENT SRG:]

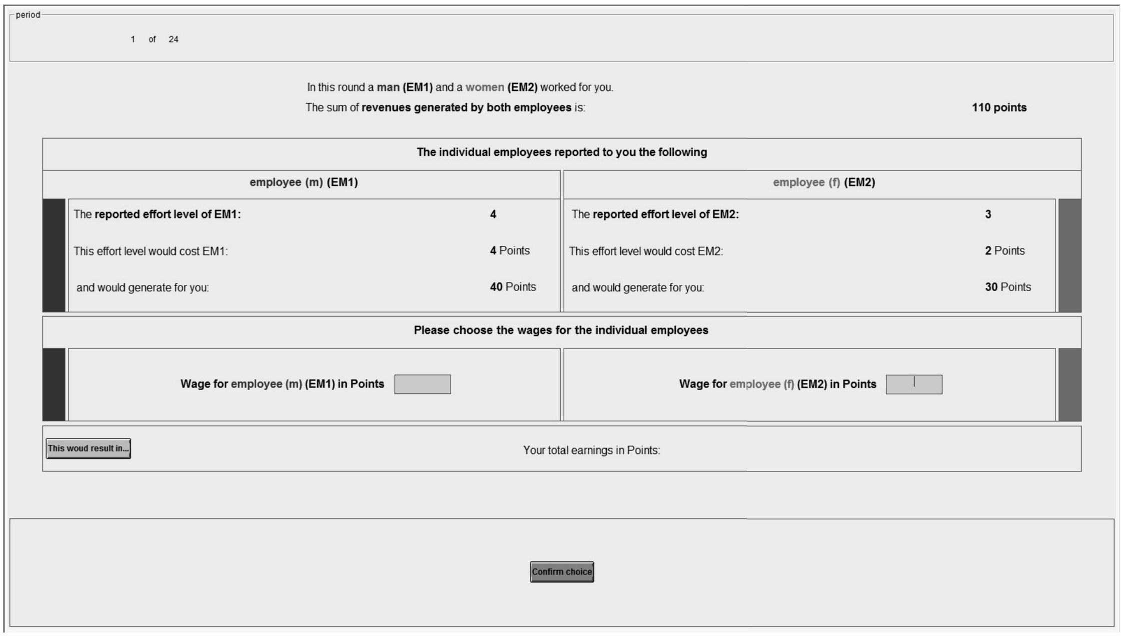

- In the upper section, **<u>the employer (m/f) is informed about employees' genders (m/f). Furthermore,</u>** total revenues generated by EM1 and EM2 in this round are displayed.

- **The middle section displays the following information to the employer <u>(m/f)</u>:**
  - **The reported efforts of stage 2 of employee 1 (EM1) and employee 2 (EM2).**
  - **The cost each employee <u>(m/f)</u> would have incurred from the reported effort. Notice that these costs do not equal the true costs when the respective employee <u>(m/f)</u> has reported an effort level different from the choice in stage 1.**
  - **The contribution to revenues each employee <u>(m/f)</u> would have made with the reported effort in stage 2. Notice that here, too, this does not necessarily equal the true contribution, which is determined by the actual effort choice of stage 1 and not the reported level.**
- In the lower section, the employer **<u>(m/f)</u>** can enter the wages for the employees **<u>(m/f)</u>** in the blue boxes. By clicking the button "this would result in…", the employer **<u>(m/f)</u>** can calculate how high his payoff would be for the wages entered. The entered choice of wages is confirmed by clicking on the red button.

Information at the end of each round

At the end of each round, employers **<u>(m/f)</u>** are informed about their total payoff from the current round and their total cumulative payoff from all rounds.

Illustration: Screen Shot End of Round Display Employer **<u>(m/f)</u>**

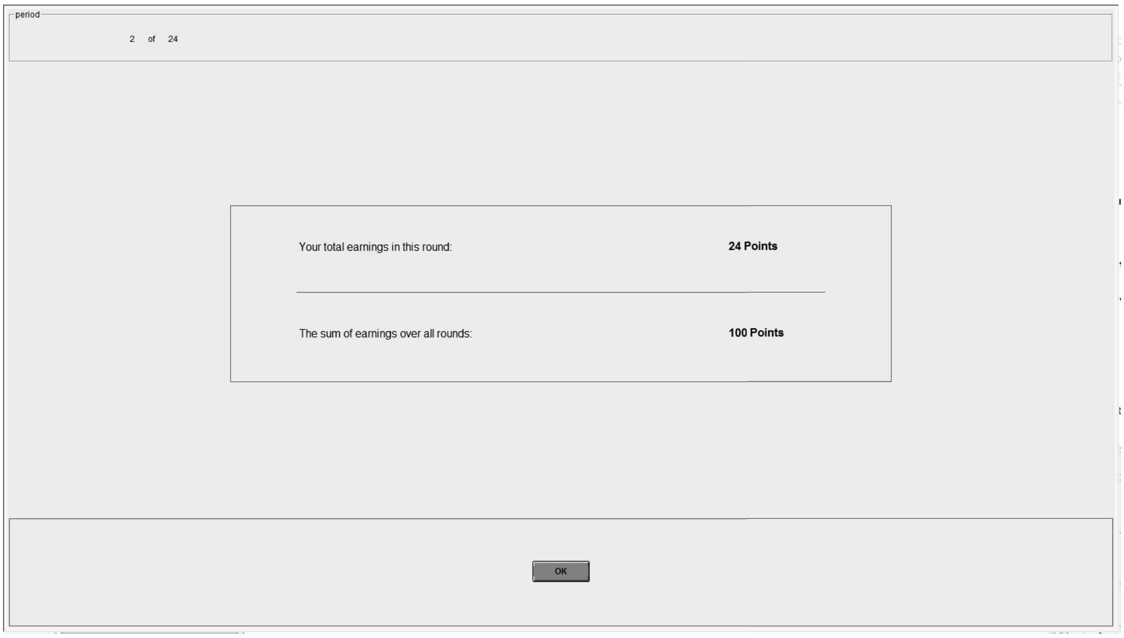

Instructions for the experiment

Illustration: Screen Shot End of Round Display Employees **(m/f)**

[TREATMENT NR:]

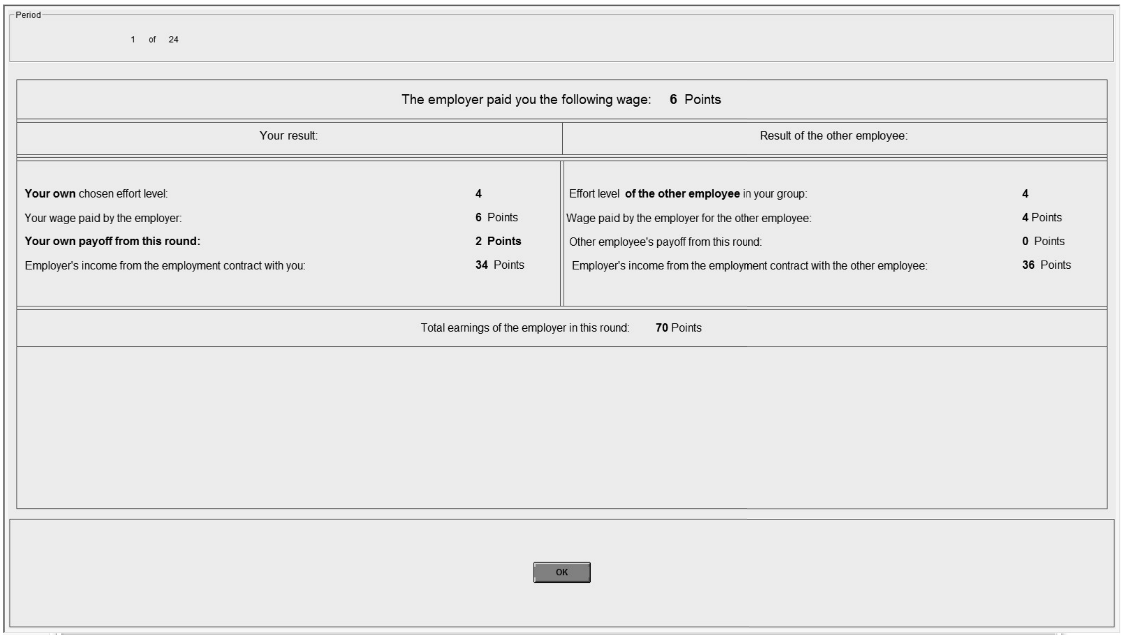

[TREATMENT SR:]

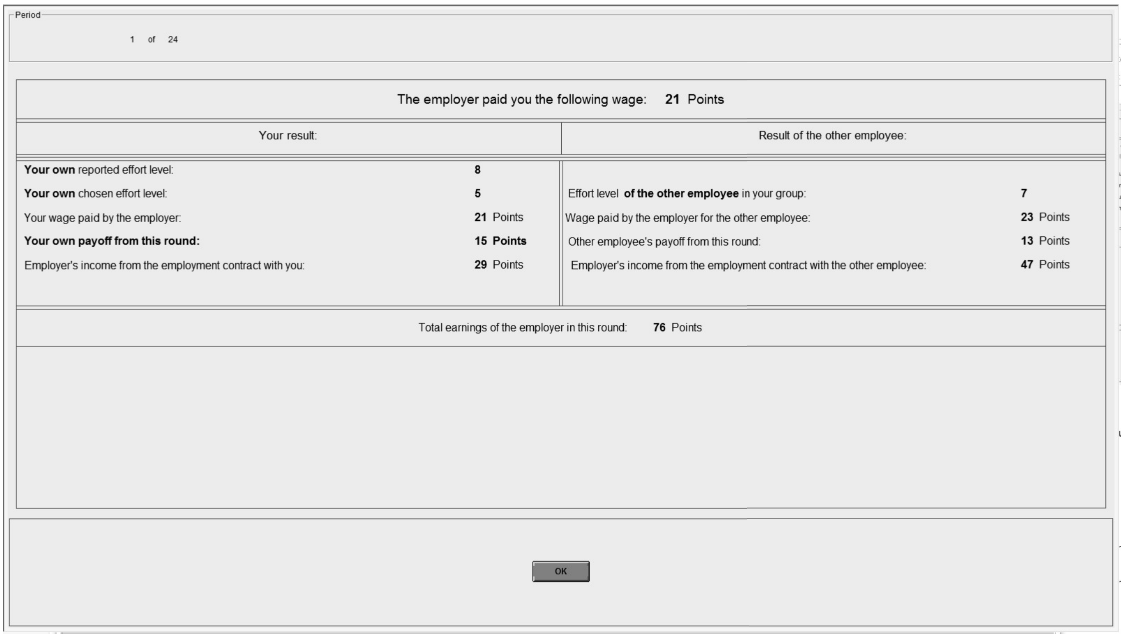

Instructions for the experiment

[TREATMENT SRG:]

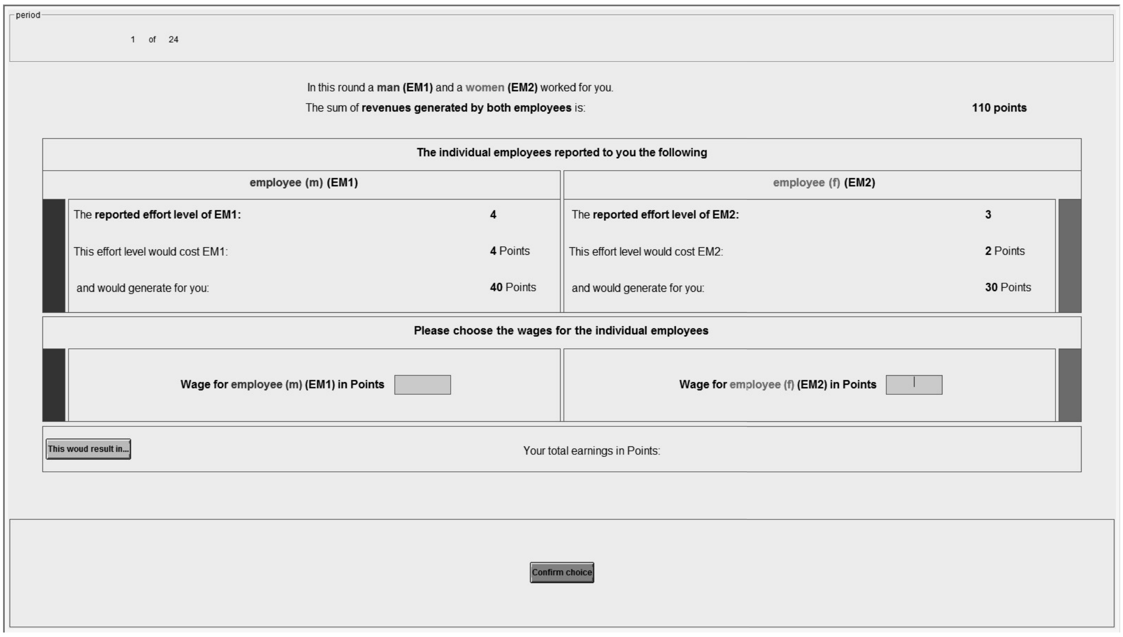

At the end of each round, employees **(m/f)** see the wage received from the employer **(m/f)** in the upper section of the screen.

The middle section contains an overview of this round's actions and payoffs in your group.

- On the left side, an employee **(m/f)** receives the following information:
  - **The own reported effort level**
  - The own chosen effort level
  - The wage paid by the employer **(m/f)**
  - The own payoff from this round
  - The own contribution to the generated revenues (employer income from the employment contract with you)
- On the right side, the employee **(m/f)** receives the following information about the other employee **(m/f)**:
  - The effort level chosen by the other employee **(m/f)**
  - The wage the employer **(m/f)** paid to the other employee **(m/f)**
  - The other employee's **(m/f)** payoff from this round
  - The other employee's contribution **(m/f)** to the generated revenues

Instructions for the experiment

Calculation of Revenues and Payoffs

Employer **(m/f)** and employee **(m/f)** payoffs in points each round are calculated as follows:

Employer **(m/f)** payoff	=	10 x effort of EM1<br>+ 10 x effort of EM 2<br>– wage for EM1 (w1)<br>– wage for EM2 (w2)
Payoff of EM 1 **(m/f)**		= wage for EM1 (w1)<br>– cost of effort of EM1
Payoff of EM 2 **(m/f)**		= wage for EM2 (w2)<br>– cost of effort of EM2

Please note once more that the reported effort does not affect revenues or effort costs.

At the end of the experiment your total earnings – base amount plus the sum of payoffs from all rounds – will be paid out to you. 1 point is worth 1.5 Cent.

If you have a question, please now raise your hand!
